# Protective Effects of Guava Pulp on Cholestatic Liver Injury

**DOI:** 10.1155/2013/601071

**Published:** 2013-11-17

**Authors:** Jian Peng, Chunyan Yue, Kai Qiu, Jie Chen, Maria-Angeles Aller, Kwang Suk Ko, Heping Yang

**Affiliations:** ^1^National Hepatobiliary and Enteric Surgery Research Center, Xiangya Hospital of Central South University, 87 Xiangya Road, Changsha, Hunan 410008, China; ^2^Surgery Department, School of Medicine, Complutense University, Madrid, Spain; ^3^Department of Nutritional Science and Food Management, The College of Health Science, Ewha Womans University, Seoul, Republic of Korea; ^4^Division of Gastroenterology and Liver Diseases, USC Research Center for Liver Diseases, Department of Medicine, Keck School of Medicine, USC, HMR Building, 415, 2011 Zonal Avenue, Los Angeles, CA 90033, USA

## Abstract

*Background*. Cholestatic liver injury is a leading cause of chronic liver diseases involved with oxidative stress changes and inflammation; thus, antioxidant and anti-inflammation compound-rich guava may play a pivotal role in protecting against the cholestatic liver damages. Our aims for this study are to determine whether guava pulp (GP) has protective effects on cholestatic liver injury-induced mouse model and on interleukin-6 (IL-6) mediated proliferation of QBC939 cholangiocarcinoma cell line. *Methods*. Mice were induced to cholestatic liver damage by left and median bile duct ligation (LMBDL) surgery and then treated with GP. Plasma and liver samples were collected for biochemical and pathological assays. 5-Bromo-2′-deoxyuridine (BrdU) assay and Western blots were used to detect proliferation and gene expression in QBC939 cells, respectively. *Results*. Compared with LMBDL only group, in GP-treated mice, the levels of alanine aminotransferase (ALT) and bilirubin decreased, biliary epithelial cell proliferation and liver fibrogenesis were suppressed, Src/MEK/ERK1/2/c-Myc pathway and expressions of transforming growth factor *β*1(TGF-*β*1), tissue inhibitor of metalloproteinases TIMP), and procollagen 1*α*1(COL1*α*1) were downregulated significantly. Moreover, the GP extract reduced IL-6-enhanced QBC939 cell proliferation, p-ERK, and c-Myc expression as well. *Conclusions*. GP may provide a new perspective for the treatment of cholestatic liver injury.

## 1. Introduction

Cholestasis, which is caused by acute or chronic interruption in bile export, is a well-known risk factor for complications after liver surgery [1]. Also, it is an important cause of liver damages. Posthepatic (obstructive) cholestasis is characterized by portal tract expansion, leukocyte infiltration, bile duct and septal proliferation, liver fibrosis, and eventually cirrhosis in human [2]. Patients with cholestasis, such as extrahepatic bile duct cancer, are at a greater risk for postoperative liver failure, sepsis, and death [3]. Therefore, intensive researches are required to find effective therapeutic agents for cholestatic liver injury.

Common bile duct ligation (CBDL) is a well known cholestatic model of extrahepatic biliary obstruction [4]. However, CBDL generally develops hepatic, intraperitoneal, and pulmonary abscesses, and even sepsis which leads to the early mortality of CBDL animals. To establish a model that is closer to the human situation, we established a new, reproducible model of chronic cholestatic liver injury [5]. In order to study chronic cholestatic liver injuries better, we also provided a detailed protocol for LMBDL surgery in this report, which might provide a patient-like environment of cholestatic liver injuries.

Guava is a plant that grows in tropical and subtropical countries. It is widely used for its multiple pharmacological activities. For example, guava leaf extract exerts hepatoprotective effects [6]. Guava fruits, which are known to contain very rich natural antioxidant compounds [7], may exert antidiabetic effect through their antioxidative and anti-inflammatory properties [8, 9]. Inflammation is important for pathologic changes that occur during LMBDL-induced cholestasis [5]. However, effects of GP on cholestatic liver injury are not widely studied. 

Inflammation-related interleukin-6 (IL-6) has been identified as a contributing factor to hepatic epithelial changes during hepatic inflammation. It is known that IL-6 exerts pleiotropic effects with both cytoprotective and mitogenic effects in biliary tract epithelia [10]; it is also known that IL-6 expression is increased in bile duct ligation model of rat [11], mice [12], cholestatic patients [13], and neonatal cholestasis [14]. Moreover, IL-6 enhanced c-Myc translation in multiple myeloma cells [15], and promoted c-Myc expression and proliferation of cultured vascular smooth muscle cells [16]. However, whether GP protects IL-6-mediated QBC939 cell proliferation and c-Myc gene expression is not determined. In this report, we examined hepatoprotective functions of GP using in vivo and in vitro cholestatic liver injury models.

## 2. Materials and Methods

### 2.1. Reagents

All other reagents were of analytical grade and obtained from commercial sources.

### 2.2. Left and Median Bile Duct Ligation (LMBDL)

Male C57BL/6 mice aged 12–14 weeks were housed in a 21 ± 2°C room on a 12 : 12 h dark-light cycle with free access to water and food. Experimental protocols were approved by the Xiangya Hospital of Central South University, Central South University, Changsha, China. We performed LMBDL to block the fluxed passages of bile in median and left lobes (approximately 70% of the liver) and removed the gallbladder to avoid cholecystitis [5]. The LMBDL surgery procedure was detailed in Supplementary Figure 1 (see Figure 1 in Supplementary Material available online at http://dx.doi.org/10.1155/2013/601071).

### 2.3. Guava Fruit and Guava Pulp Extraction

**Figure 1:**
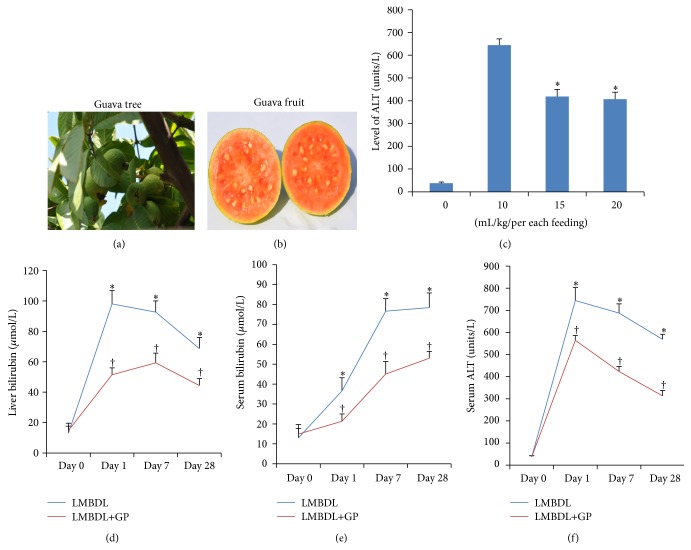
Effect of guava pulp on bilirubin and alanine aminotransferase (ALT) levels. (a) The guava tree. The guava tree is the apple guava (*Psidium guajava*) tree. It is native to Mexico. (b) Guava fruit. Guava fruit is oval, with a soft and sweet taste. As guava becomes mature, the skin changes into yellow from green, with deep pink fresh (“red” guavas) and the seeds in the central pulp of variable number and hardness. (c) Dose response of GP on serum ALT level in the LMBDL. The mean ± standard errors are 37.5 ± 5.4, 644.2 ± 27.6, 418.6 ± 31.5, and 407.1 ± 30.2 in the groups of 0, 10, 15, and 20 (mL/kg/per each feeding). ^*^ *P* < 0.01 10 mL/kg/per each feeding versus 15 and 20 mL/kg/per each feeding, respectively. (d) Bilirubin levels in the ligated and unligated lobes. Liver homogenate was extracted from the ligated (left and median) and unligated (right and caudate) lobes to assess changes of bilirubin level. ^*^ *P* < 0.01, the ligated lobes at day 1, 7 and 28 versus the unligated lobes at days 1, 7, and 28, respectively. (e) Serum bilirubin level. ^*^ *P* < 0.05, the LMBDL+GP groups at days 1, 7, and 28 versus the LMBDL groups at 1, 7, and 28, respectively. (f) Serum alanine aminotransferase (ALT) levels. ^*^ *P* < 0.01, ^*^ *P* < 0.05 the LMBDL+GP groups at days 1, 7, and 28 versus the LMBDL groups at 1, 7, and 28, respectively.

Guavas were sorted (Figures 1(a) and 1(b)), washed, and then crushed in a blender. Fresh pure GP juice was stored in 10 mL tubes, frozen in −20°C refrigerator, and thawed before gavage feeding. After gavage feeding, remainder was thrown away. 

For GP extraction, guava fruits were flushed by tap water then washed in distilled water for three times, screw-capped, and cut into small pieces before being dried in a hot air-blowing oven at 50°C. The GPs were ground to a very fine powder in a blender and kept in refrigerator prior to extraction and were extracted according to the method of Bontempo et al. [17]. 10 g dry powder was extracted with 100 mL of 70% alcohol in a screw-capped guava pulp and shaken at room temperature for 24 h. The extracts were centrifuged at 5000 g for 10 min, and the residue was extracted again under the same conditions twice and filtered with filter paper (Whatman no. 1). The 70% alcohol extractions were concentrated under low pressure, lyophilized to obtain powders, and stored at 4°C before assay.

### 2.4. Animal Groups, Diets, and Euthanasia

A standard chow was fed to all mice throughout the study (3.79 kcal/g, with 24% energy derived from protein, 12% from fat, and 64% from carbohydrate). To determine the dose-response protective effects of GP on liver damage by LMBDL, we tested ALT level at three doses of 10, 15, and 20 mL/kg/BW GP juice. Since 15 mL/kg/BW GP juice had the best beneficial effect on serum level of alanine aminotransferase (ALT) in the pilot experiments (Figure 1(c)), the dose was applied for treatment of LMBDL mice. For LMBDL, the mice were divided into 2 groups (8 per group) and given the following treatments: group 1, LMBDL plus 0.9% saline solution gavage and group 2, LMBDL plus GP gavage. The mice from each group were examined at day 0, 1, 7, and 28 after the start of the treatments. All animals were checked for body weight, activity, and jaundice daily from day 1 to day 7 after the LMBDL procedure and every other day from week 2. Analyses were performed on day 0, 1, 7 and 28 according to phase of LMBDL-induced liver injury in the short (8–48 h), intermediate (3–7 days), and long (14–45 days) term [18]. After treatments, mice were deeply anesthetized by intraperitoneal sodium pentobarbital (45 mg/kg), and blood samples were quickly obtained by cardiac puncture of the right atrium. Serum was then obtained at speed of 120 g for 15 min at room temperature and stored individually at −80°C before biochemical analyses.

### 2.5. Measurement of Alanine Aminotransferase and Bilirubin

Bilirubin (Thermo Electron, WALTham, MA) and alanine aminotransferase (ALT) (RAICHEM, San Marcos, CA) levels in serum and liver tissue were measured following the manufacturers' instruction.

### 2.6. Histology and Immunohistochemistry

Paraffin embedded liver sections were stained with H&E or Sirius red using standard histological techniques. Liver fibrogenesis was analyzed by staining with 0.1% Sirius Red (Sigma, St. Louis, MO), quantified using a computer-assisted image analysis system (MetaMorph imaging system; Universal Imaging, Downingtown, PA), and expressed as stained area per total examined area as previously described [19]. In addition, sections were immunostained for c-Myc, Ki-67, and CK19 assays (Abcam, Cambridge, MA). Proliferation of cholangiocytes was assessed in liver sections from the treatment groups by (1) immunohistochemical staining for CK19 (to assess intrahepatic biliary mass) and (2) PCNA immunoreactivity as a marker of proliferative capacity [20]. Ten small portal fields were chosen per sample, and the number of bile ductules was counted.

### 2.7. Gene Expression Assays

Mouse liver samples were homogenized in Trizol (Invitrogen, Carlsbad CA) to extract total RNA, which was then purified using RNA easy minicolumn and on-column, digested with DNase I (Qiagen, Valencia CA), and reverse-transcribed into cDNA using SuperScript II RNase H Reverse Transcriptase (Invitrogen, Carlsbad, CA). Quantitative real-time PCR was performed in duplicate. 9 *μ*L cDNA was mixed with 10 *μ*L 2 × TaqMan universal master mix and 1 *μ*L custom 20 × TaqMan primer and probe mix (Applied Biosystems, Foster City, CA) for mouse procollagen 1*α*1 (assay ID Mm00801666_g1), tissue inhibitor of metalloproteinase 1 (assay ID Mm00446231_m1), transforming growth factor *β*1 mRNA (assay ID Mm00441818_m1), and glyceraldehyde-3-phosphate dehydrogenase (assay ID Mm99999915). The following PCR conditions were used: 50°C for 2 min, 95°C for 10 min, followed by 39 additional cycles at 95°C for 15 s, and 60°C for 1 min. The expression level of each target gene was normalized with GAPDH.

### 2.8. Effect of Guava Pulp Extraction on QBC939 Cell Proliferation

We have created animal model with human-like environment for systematic research, and for microenvironment research, the human cholangiocarcinoma cell line QBC939 was purchased from ATCC. The cell was cultured under the following condition: RPMI 1640 containing 10% FBS, 37°C, and 5% CO_2_. To determine the effect of GP extraction on QBC939 cell proliferation, 2 × 10^4^ cells were plated on a 24-well plate. After 48 h, the medium was changed to serum-free RPMI 1640 medium, and the cells were incubated for an additional 24 h to deplete endogenous steroid hormones prior to experiments. Cells were then treated with GP extraction of different concentrations (0, 0.1, 0.2, 0.4, 0.8, and 1.6 mg/mL) and cultured for another 48 h. Bromodeoxyuridine (BrdU) was added to each well for 4 hours and measured using the BrdU Cell Proliferation Assay Kit (CalBiochem, San Diego, CA).

### 2.9. Proteome Profiler and Western Blot

The phosphor-receptor tyrosine kinase (phosphor-RTK) array was purchased from R&D Systems (Cat# ARY001, Minneapolis, MN) and performed following the kit instructions. Cytoplasmic protein was isolated from the liver tissues and cell culture as previously described [5]. Western blot analysis was done using antibodies to phosphor-ERK1/2, phosphor-JNK, phosphor-MEK1/2, phosphor-Src, and c-Myc (Cell Signaling, Danvers, MA).

### 2.10. Data Analysis

Data were given as mean ± standard error. Statistical analysis was performed using analysis of variance followed by Fisher's test for multiple comparisons. Statistic significance was defined as *P* ≤ 0.05.

## 3. Results

### 3.1. Effect of Guava Pulp on Cholestatic Liver Damage

To better determine the effects of GP on cholestatic liver injury, we performed LMBDL to create cholestatic liver injury model [5] and described it in Supplementary Figures 1(a)–1(i). Animals treated with LMBDL and LMBDL+GP showed a sharp increase in bilirubin levels of the ligated lobes at day 1, remained at high levels for up to day 7, and then decreased at day 28. Even on day 28, the bilirubin in the ligated lobes of LMBDL or LMBDL+GP groups was 10.7- and 5.5-fold higher than day 0, respectively (Figure 1(d)). The bilirubin in the unligated lobes of LMBDL and LMBDL+GP groups on day 28 was 8.4- and 3.4-fold higher than day 0, respectively (Figure 1(d)). Even though GP feeding could not recover bilirubin levels of the ligated lobes to normal, it significantly decreased bilirubin levels in the ligated lobes.

The serum bilirubin levels in LMBDL and LMBDL+GP groups were increased from day 1 to 7. It was 5.8- and 3.6-fold higher in day 28 than day 0 (Figure 1(e)). For the LMBDL or LMBDL+GP mice, there was an acute increase in ALT levels of serum at day 1 but steadily decreased thereafter (Figure 1(f)). At day 28, the ALT levels in the LMBDL and LMBDL+GP were 15.1- and 6.3-fold higher than day 0, respectively. The levels of serum bilirubin and ALT in LMBDL groups were decreased significantly by the addition of GP feeding (Figures 1(d)–1(f)). 

### 3.2. Effect of Guava Pulp on Cholestatic Liver Injury and Biliary Epithelial Cell Proliferation

**Figure 2:**
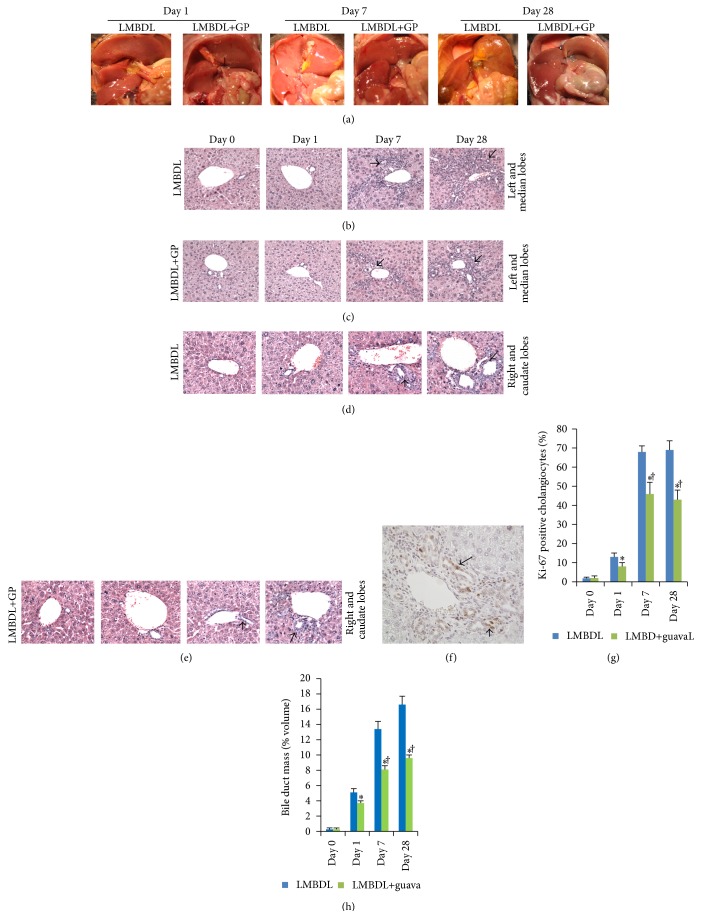
Effect of guava pulp on hepatobiliary system and biliary epithelial cell proliferation. (a) The hepatobiliary system at days 1, 7, and 28. Progressive liver injury appeared at LMBDL groups from days 1 to 28. Representative H&E (×200) results of the ligated livers from the LMBDL group (b), the ligated livers from the LMBDL+GP group (c), the unligated livers from the LMBDL (d), and the unligated livers from LMBDL+GP (e), respectively. Arrow (↑)  =  biliary epithelial cell proliferation. (f) Representative Ki-67 immunohistochemical results of the proliferative ductules in the LMBDL group at day 28. Arrow  =  Ki-67 positive biliary epithelial cells. ((g)-(h)) Quantification of Ki-67 positive biliary epithelial cells and bile duct mass. Ki-67 and CK-19 results of biliary epithelial cells in the left and median lobes were evaluated for proliferation. ^*^ *P* < 0.01, the liver tissues from the sham operation versus the ligated lobes in LMBDL and LMBDL+GP groups. ^†^ *P* < 0.01, the ligated lobes of LMBDL at day 1 versus the ligated lobes at days 7 and 28.

We examined the changes of the hepatobiliary system after day 1, 7, and 28 after surgery. Animals treated with LMBDL showed more progressive liver injury than mice treated with LMBDL+GP (Figure 2(a)). One of the characteristic changes in LMBDL was conspicuous BEC proliferation in the ligated lobes (Figure 2(b)), while GP feeding reduced the BEC proliferation (Figure 2(c)). Moreover, there was a slight increase in BEC proliferation at the unligated lobes (Figure 2(d)), while GP administration reduced the BEC proliferation (Figure 2(e)). Ki-67 positive BECs were shown in Figures 2(f) and 2(g) and expressed as a percentage of all BECs. For the LMBDL and LMBDL+GP mice, there was an increase of Ki-67 positive cells after day 1 but maintained at high levels thereafter. At day 28, the Ki-67 positive cells in the LMBDL and LMBDL+GP groups were 38.2- and 22.5-fold higher than day 0, respectively (Figure 2(g)). Bile duct mass was steadily increased from day 1 to 28 after LMBDL and was reduced by GP, as demonstrated by CK-19 immunoreactivity (Figure 2(h)). Thus, GP can reduce LMBDL-induced BECs proliferation. 

**Figure 3:**
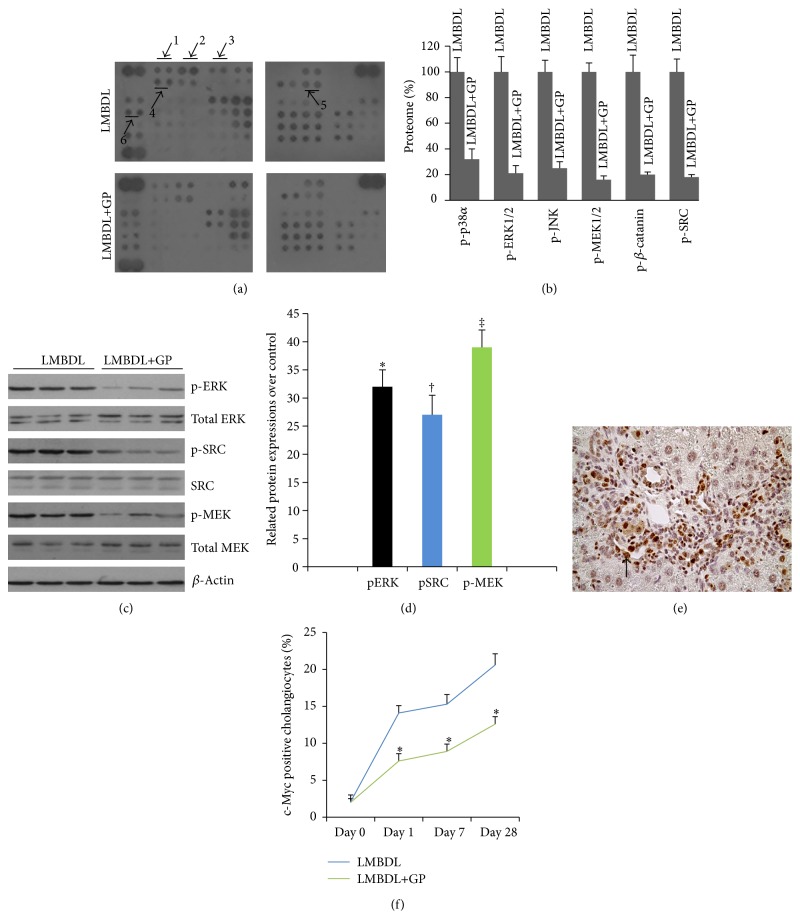
Effect of guava pulp on gene expression. (a) The phosphor-receptor tyrosine kinase (Phosphor-RTK) array. Cytoplasmic proteins from the ligated lobes in LMBDL and LMBDL + guava groups. Arrow no. 1, no. 2, no. 3, no. 4, no. 5, and no. 6 represented phosphor-p38*α*, phosphor-ERK1/2, phosphor-JNK, phosphor-*β*-catenin and phosphor-Src, respectively. (b) Densitometric analysis of p-p38*α*, pERK, pMEK, pJNK, p-*β*-catenin, and pSrc in the ligated lobes of LMBDL and LMBDL + guava. (c) Representative Western blots for the expression level of pERK, total ERK, pMEK, total MEK, pSrc, and total Src in the ligated lobes in LMBDL and LMBDL+GP groups. (d) Quantitative assays of densitometric changes expressed as percentage of phosphorylation over total protein. A total of 24 animals (6 per group) were studied at day 28. ^*^ *P* < 0.01, percentage of pERK/total ERK in LMBDL+GP group versus percentage of pERK/total ERK in LMBDL group, ^†^ *P* < 0.01, percentage of pSrc/total Src in LMBDL+GP group versus percentage of pSrc/total Src in LMBDL group, ^‡^ *P* < 0.01, percentage of pMEK/total MEK in LMBDL+GP group versus percentage of pMEK/total MEK in LMBDL group. (e) Determination and quantification of c-Myc positive biliary epithelial cells. ^*^ *P* < 0.01 the ligated lobes of LMBDL+GP groups at days 1, 7, and 28 versus the ligated lobes of LMBDL groups at 1, 7, and 28, respectively.

Mechanisms regulating BECs proliferation in cholestatic liver diseases are poorly understood. To further elucidate this important issue, we examined relative levels of phosphorylation of 46 kinase phosphorylation sites using Phospho-Kinase Array. At day 28, animals treated with LMBDL+GP resulted in a 3.1-, 4.8-, 4.0-, 6.3-, 5.0-, and 5.6-fold falls in ratios of phosphor-p38*α*, phosphor-ERK1/2, phosphor-JNK, phosphor-MEK1/2, phosphor-*β* catenin, and phosphor-Src compared with the LMBDL, respectively (Figures 3(a) and 3(b)). One of the candidate mechanisms is that phosphorylation of ERK is involved in BECs proliferation [21]. The Src/ERK pathway plays an important role in the regulation of BECs growth and secretion in cholestatic liver diseases [22]. So, we focused on the changes of phosphor-ERK, phosphor-Src and phosphor-MEK. Western blot confirmed that phosphor-ERK1/2, phosphor-Src, and phosphor-MEK expressions in the LMBDL+GP group accounted for 32%, 27%, and 39% of the total ERK, Src, and MEK expressions, respectively, which were 2.1-, 2.7-, and 1.6-fold lower than the LMBDL group, respectively (Figures 3(c) and 3(d)).

We have already reported that aberrant c-Myc expression is associated with BECs proliferation [5]. ERK is implicated in the regulation of c-Myc expression [23]. c-Myc positive cell staining was shown in Figures 3(e) and 3(f). We found that c-Myc expression increased at day 1, peaked at day 7, and maintained high level at day 28 in LMBDL groups (Figures 3(e) and 3(f)). Animal treated with LMBDL+GP could reduce the expression of c-Myc from day 1 to day 28 (Figure 3(f)). Our results indicated that activation of Src/MEK/ERK1/2/c-Myc pathway was consistent with BECs proliferation in LMBDL groups, while LMBDL+GP reduced activation of Src/MEK/ERK1/3/c-Myc pathway and inhibited BECs growth. 

### 3.3. Effect of Guava Pulp on Liver Fibrogenesis

**Figure 4:**
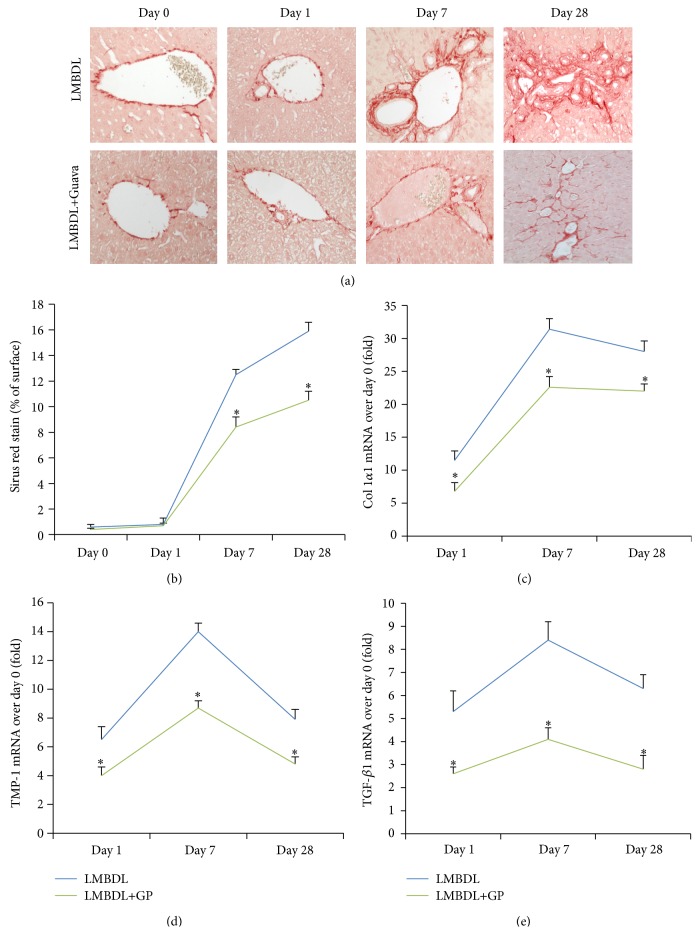
Effects of guava pulp on liver fibrogenesis, relative gene expression. (a) Representative pictures of liver fibrogenesis at days 1, 7, and 28 following LMBDL (top row) and LMBDL+GP (bottom row) by Sirius Red stain (200x). (b) Percentage of liver surface stained with Sirius red. Values were mean (s.e.m.) for five to six individual animals per time point. ^*^ *P* < 0.01, the ligated lobes of LMBDL versus LMBDL+GP at days 1, 7, and 28, respectively. ((c)–(e)) Levels of procollagen 1*α*1, metalloproteinases 1 and transforming growth factor *β*1 mRNA are determined by reverse transcriptase-polymerase chain reaction and expressed as fold induction in comparison to sham-operated controls. Values were mean (s.e.m.) for five to six individual animals per time point. (c) Expression of procollagen 1*α*1. ^*^ *P* < 0.01, the ligated lobes of LMBDL+GP groups at days 1, 7, and 28 versus the ligated lobes of LMBDL groups at days 1, 7, and 28, respectively. (d) Expression of metalloproteinases 1. ^*^ *P* < 0.01 the ligated lobes of LMBDL groups at days 1, 7, and 28 versus the ligated lobes of LMBDL+GP groups at days 1, 7, and 28, respectively. (e) Expression of TGF-*β*1. ^*^ *P* < 0.01, the ligated lobes of LMBDL at days 1, 7, and 28 versus the ligated lobes of LMBDL+GP at days 1, 7, and 28, respectively.

Biliary epithelial cells (BECs) provide the first line of defense against lumenal microbes in the biliary system, and it might play a key role in the progression of cholangiofibrosis during cholestatic liver diseases, such as primary biliary cirrhosis (PBC) and primary sclerosing cholangitis (PSC) [24]. Interestingly, we found that BEC proliferation was consistent with liver fibrogenesis caused by cholestatic liver injury (Figure 4(a)). To determine whether GP has protective effects against liver fibrogenesis, we performed quantification of the Sirius red-positive liver surface with or without GP treatment. Mice subjected to LMBDL showed that collagen accumulation appeared at day 1, further increased at day 7, and severe accumulation of collagen was observed until day 28, while LMBDL with GP treatment significantly suppressed the liver fibrogenesis from day 1 to 28 (Figures 4(a) and 4(b)).

Procollagen 1*α*1 (COL1*α*1), tissue inhibitor of metalloproteinase-1 (TIMP-1), and transforming growth factor *β*1 (TGF-*β*1) are markers for liver fibrogenesis [18]. Quantitative RT-PCR was used to determine the time course of the three genes expression. As shown in Figure 4(d), the COL1*α*1 mRNA level in LMBDL and LMBDL+GP on day 1 was 11.5- and 6.8-fold higher compared with day 0, respectively. COL1*α*1 mRNA level peaked in day 7, which were 31.4- and 22.6-fold higher in LMBDL and LMBDL+GP groups compared with day 0, respectively. At day 28, the COL1*α*1 level in the LMBDL and LMBDL+GP groups was 28- and 22-fold higher compared with the day 0, respectively. 

The TIMP-1 mRNA level in LMBDL and LMBDL+GP on day 1 was 6.5- and 4-fold higher compared with day 0, respectively. TIMP-1 mRNA level peaked in day 7, and it showed 14- and 8.7-fold higher in LMBDL and LMBDL+GP groups compared with day 0, respectively. At day 28, the TIMP1 level in the LMBDL and LMBDL+GP groups was 7.9- and 4.8-fold higher compared with the day 0, respectively (Figure 4(d)). 

The TGF-*β*1 mRNA level in LMBDL and LMBDL+GP on day 1 was 5.3- and 2.6-fold higher compared with day 0, respectively. TGF-*β*1 mRNA level peaked in day 7, and it was 8.1- and 4.1-fold higher in LMBDL and LMBDL+GP groups compared with day 0, respectively. At day 28, the TGF-*β*1 level in the LMBDL and LMBDL+GP groups was 6.3- and 2.8-fold higher compared with the day 0, respectively (Figure 4(e)). Therefore, GP treatment could effectively reduce COL1*α*1, TIMP-1, and TGF-*β*1 mRNA expression compared with LMBDL alone. 

### 3.4. Effect of Guava Pulp on IL-6-Mediated QBC939 Cell Proliferation and Relative Gene Expression

**Figure 5:**
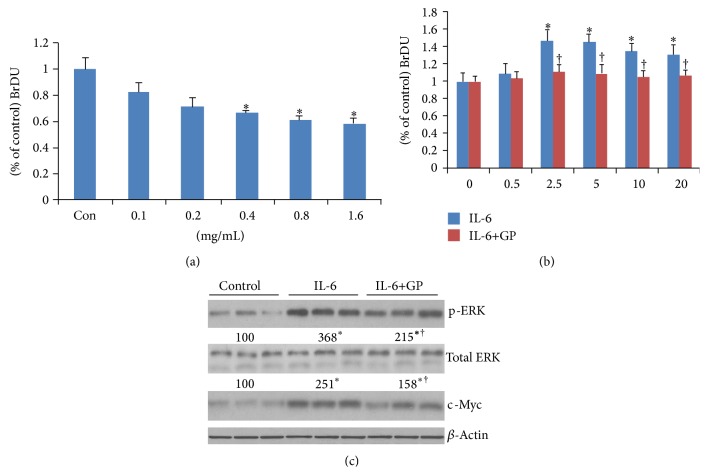
Effect of guava pulp on QBC939 cell growth, p-ERK, and c-Myc gene expression. (a) BrdU assay of QBC939 cells after treatment with *P. guajava* L. pulp extract for 24 h. ^*^ *P* < 0.01, the groups at concentration of 0.4 mg/mL, 0.8 mg/mL and 1.6 mg/mL versus control. Con = control (0 mg/mL). (b) QBC939 cells treated with IL-6 and GP. QBC939 cells were treated with IL-6 at concentration of 0.5, 5, 10, 20 and 40 (ng/mL) for 24 h. ^*^ *P* < 0.05, control (Con) versus groups of 2.5, 5, 10, and 20 IL-6 (ng/mL) treatment, respectively. GP treatment significantly reduced IL-6-induced QBC939 cell proliferation. ^†^ *P* < 0.05,  IL-6 treatments versus IL-6+GP treatment. Proliferation was assessed using BrdU cell proliferation assay. (c) Representative Western blots for the expression level of pERK and c-Myc at 24 h with or without treatment with GP extract and IL-6. Quantitative assays of densitometric changes expressed as percentage of phosphorylation over total protein QBC939 cells at 24 h after treatment with GP extract (0.8 mg/mL). ^*^ *P* < 0.01, control versus IL-6 or IL-6+GP treatment, ^†^ *P* < 0.01,  IL-6 treatment versus IL-6+GP treatment.

Bontempo et al. reported that GP extract exerted antineoplastic effects through induction of apoptosis and cell differentiation in acute promyelocytic leukemia cell line NB4 [17]. Our data have shown that LMBDL induces with BECs proliferation [5], while GP can lower LMBDL-induced BECs proliferation (Figures 2(b) and 2(c)). IL-6 is an important proinflammatory cytokine during cholestatic liver injury. To assess the role of GP in IL-6-mediated QBC939 cells in vitro, BrdU was used to determine the cell proliferation. GP treatment decreased IL-6-mediated QBC939 proliferation (Figures 5(a) and 5(b)). What is more, compared with control group, p-ERK expressions in IL-6-mediated group and IL-6-mediated + GP group were increased to 3.68- and 2.15-fold, respectively, and c-Myc expressions were increased to 2.15- and 1.68-fold, respectively. The results indicate that GP can downregulate p-ERK and c-Myc expressions, and the reduction of QBC939 proliferation in the GP treatment was consistent with downregulation of p-ERK and c-Myc expression (Figure 5(c)).

## 4. Discussion

Chronic cholestatic liver diseases are a leading indication of liver transplantation in adults and children [25, 26], and genetic defects, mechanical aberrations, toxins, and/or dysregulations in the immune system cause the bile duct damage and accumulation of bile [27]. Then, the accumulation of potentially toxic bile acids (BAs) leads to hepatocellular damage followed by inflammation and fibrosis and, finally, depending on the disease severity and duration, may culminate in liver cirrhosis and hepatocellular or cholangiocellular cancer requiring liver transplantation. Great progress has been made in the last decade in our understanding of the molecular basis of bile formation and the pathobiology of cholestasis [28, 29]. However, there is no medical treatment with proven efficacy for patients with cholestasis [30]. Thus, it is necessary to find novel therapeutic agents for chronic cholestatic liver diseases.

Guavas (*Psidium guajava* L.) have been long recognized as an important economical fruit. Several studies have proved that GP extract from the fruit, leaf, bark, or roots of *psidium guajava *had potential pharmacological activities manifested as antioxidant, hepatoprotective, and anti-inflammatory properties [31]. *Psidium guajava *fruit peel aqueous extract at dose of 400 mg/kg produced significant hepatoprotection to rat liver damage induced by carbon tetrachloride [6]. The guava fruit is a very good source of flavonoids such as *β*-carotene, lycopene, lutein and cryptoxanthin, and vitamins A and C. These compounds are known to have antioxidant properties and are essential for appropriate heALTh. We proposed the possibility of the protective activity of GP against cholestatic liver damage. Based on these findings of multiple effects of GP, our present study aimed to examine the protective activity of GP against cholestatic liver damages. We applied LMBDL model of mice to induce cholestatic liver damage (Supplementary Figures 1(a)–1(i)) and assessed the potential protective activity of GP. We found that LMBDL resulted in elevation of bilirubin and ALT (Figures 1(c) and 1(d)), which can cause toxicity to cholangiocytes and other hepatic cells. Interestingly, there were the effects of guavas for anticholestasis and antiliver injury (Figures 1(c), 1(d), and 2(a)–2(e)). 

BECs proliferation is observed in all human cholestatic liver diseases. In the LMBDL and LMBDL+GP mice, there was an increase of BECs proliferation at day 1 but maintained high levels thereafter. GP can effectively reduce LMBDL-induced BECs proliferation (Figures 2(a)–2(e)). What are the mechanisms regulating BECs proliferation in cholestatic liver diseases? It has been reported that phosphorylation of ERK is related to BECs proliferation [21]. We analyzed phosphorylation patterns in LMBDL and LMBDL+GP. Results showed that animal treated with LMBDL increased expression in p-ERK1/2, p-MEK1/2, and p-Src compared with the LMBDL+GP (Figures 3(a)–3(d)). The Src/MEK/ERK1/2 pathway plays an important role in the regulation of BECs growth observed in cholestatic liver diseases [21]. The MEK/ERK pathway is involved in the regulation of proteolytic degradation and stability of c-Myc [23]. We found that animal treated with GP can reduce the expression of c-Myc, and that GP-reduced cholestatic liver injury might be relative to downregulation of Src/MEK/ERK1/2/c-Myc pathway (Figures 3(c)–3(f)).

Liver fibrogenesis begins with an early proliferation of BECs and portal periductular fibroblasts [32]. TGF-*β*1 plays an important role in liver fibrogenesis through acting on matrix-producing cells [33] its during progress it stimulates procollagen 1*α*1, which is the most common fibrous form [34]. TGF-*β*1 also enhanced the expression and secretion of TIMP-1 [35], which is a fibrogenesis marker. The increased synchronization of all indices with cellular proliferation in our study has demonstrated the tight correlation between ductular proliferation, liver fibrogenesis, and aberrant expression of TGF-*β*1, TIMP-1, and COL1*α*1 (Figures 4(a)–4(e)). 

Oxidative stress occurs in the cholestatic liver injury [19, 36]. Chronic inflammation via pro-inflammatory cytokines (i.e., IL-6) and transcription factor NF-*κ*B controls oxidative stress response of the enzymes cyclooxygenase 2 (COX-2) and inducible nitric oxide synthase (iNOS) to generate reactive oxygen species (ROS) and reactive nitrogen species (RNS) which disturb homeostasis of many adaptive response systems such as oxidant/antioxidant ratio, DNA repair enzymes including many ALTered candidate genes involvement in cell proliferation, apoptosis, and fibrogenesis [37]. Overproduction of ROS and RNS results in genotoxic DNA damage in the opisthorchiasis-induced cholangiocarcinoma (CCA) [37]. Moreover, excess ROS and RNS can increase endogenous nitrosation reactions to yield carcinogenic N-nitrosamines. Both IL-6 and N-nitrosamines lead to c-Myc expression. Guava is rich in polyphenolic antioxidative and anti-inflammatory compounds such as tannins, phenolics, and flavonoids [38]. Polyphenolic compounds have been shown to downregulate c-Myc gene expression in Caco-2 cell [39], ovarian cancer cells [40], and human embryonal kidney cells [41]. So far, the hepatoprotecting benefits of guava have been limited. Studies show that guava extract reduces cancer risk through its antioxidant activities and by inducing apoptosis in prostate cancer cell line LNCaP [42], and it has also been shown to be highly reactive toward oxygen free radicals [43]. Our preliminary data demonstrated that GP on IL-6-mediated growth inhibition of CCA cell line QBC939 is correlated with downregulation of c-Myc (Figures 5(a)–5(c)). We found that GP could reduce IL-6-mediated p-ERK expression (Figure 5(c)). 

*Psidium guajava *is a plant belonging to the family Myrtaceae. At present, many previous studies focused on the effects of guava leaf, which was not edible for most consumers. However, the guava fruit and its products such as juice are extensively consumed; thus, future research in the fruit, rather than the leaves, would be helpful. The fruits, leaves, and bark of guava have been used in herbal medicines, and they exhibit many therapeutic effects including anti-inflammation. Some investigators suggested that the active components in guava fruits are oleanolic acid, ursolic acid, glucuronic acid and arjunolic acid, flavonoids: guaijavarin and quercetin, and saponin combined with oleanolic acid: morin-3-O-*α*-L-lyxopyranoside and morin-3-O-*α*-larabinopyranoside and pentane-2-thiol [44]. Thus, it is clear that *P. guajava* contains many components reported to display efficacy against aberrant proliferation. Antiproliferation of guava has been investigated in our study. Even though a variety of constituents is present in the fruit pulp extracts, the main ones are bioflavonoids. Crude acetone extract was able to reduce BECs growth in vivo and proliferation in QBC939 cells. This confirmed our hypothesis that guava would have protective roles in cholestatic liver injury and cholangiocarcinoma cell proliferation. 

## Supplementary Material

Supplementary Figure: Procedure of LMBDL surgery. A) Ketamine (100 mg/kg/BW) with Xylazine (5 mg/kg/BW) was intraperitoneally injected into the lower right quadrant of the mouse. B) Hair was removed from the operative site. C) The mouth of the mouse was gently prying opened with forceps, and then the tongue was pulled out and held to the right side. D) The abdomen skin was cleaned by swabbing with 70% ethanol solution followed by povidone iodine solution to prevent bacterial infection during the LMBDL surgical procedure. Then, laparotomy was performed to expose the abdominal contents with a small pair of scissors. The incision about 3 cm long started at the midabdomen and ended at the xiphoid process. E) Two pieces of gauze were moistened (sterile 0.9% saline) and placed on the right side of the incision (left side of mouse). Three pieces of moistened cotton gauzes were used to lift the left lobe onto the skin of the left abdomen. F) The median lobe onto the skin of the right abdomen. G) The bile duct and associated structures were exposed. Upper margin of the right lobe was determined and the portal triad (bile duct, portal vein and hepatic artery) was revealed carefully. The key step was isolation of the hepatic bile duct between the mergence of the left and median lobe and the mergence of the right and caudate lobe. H,I) The isolated hepatic bile duct was ligated with a 6-0 silk suture. Since the fluxed passages of bile in median and left lobes (approximately 70% of the liver) were blocked, the ligated bile duct would soon be full of bile. After the isolated bile duct to the gall bladder was ligated, the gallbladder was removed to avoid cholecystitis. The peritoneum was closed with a 6-0 silk suture.

## Abbreviations

ALT: Alanine aminotransferase
BECs: Biliary epithelial cells
BrdU: 5-Bromo-2′-deoxyuridine
CCA: Cholangiocarcinoma
COL1*α*1: Procollagen 1*α*1
GP: Guava pulp
IL-6: Interleukin-6
JNKs: c-Jun N-terminal kinases
LMBDL: Left and median bile duct ligation
PBC: Primary biliary cirrhosis
PSC: Primary sclerosing cholangitis
ROS: Reactive oxygen species
RNS: Reactive nitrogen species
RT-PCR: Reverse transcriptase-polymerase chain reaction
TGF-*β*1: Transforming growth factor *β*1
TIMP: Tissue inhibitor of metalloproteinases.

## References

[1] Clavien P., Petrowsky H., DeOliveira M. L., Graf R. (2007). Strategies for safer liver surgery and partial liver transplantation. *The New England Journal of Medicine*.

[2] Li M. K., Crawford J. M. (2004). The pathology of cholestasis. *Seminars in Liver Disease*.

[3] Schroeder R. A., Marroquin C. E., Bute B. P., Khuri S., Henderson W. G., Kuo P. C. (2006). Predictive indices of morbidity and mortality after liver resection. *Annals of Surgery*.

[4] Yang H., Li T. W. H., Ko K. S., Xia M., Lu S. C. (2009). Switch from Mnt-Max to Myc-Max induces p53 and cyclin D1 expression and apoptosis during cholestasis in mouse and human hepatocytes. *Hepatology*.

[5] Yang H., Li T. W. H., Peng J. (2011). A mouse model of cholestasis-associated cholangiocarcinoma and transcription factors involved in progression. *Gastroenterology*.

[6] Rai P. K., Mehta S., Watal G. (2010). Hypolipidaemic & hepatoprotective effects of *Psidium guajava* raw fruit peel in experimental diabetes. *Indian Journal of Medical Research*.

[7] Akinmoladun A. C., Obuotor E. M., Farombi E. O. (2010). Evaluation of antioxidant and free radical scavenging capacities of some Nigerian indigenous medicinal plants. *Journal of Medicinal Food*.

[8] Huang C., Yin M., Chiu L. (2011). Antihyperglycemic and antioxidative potential of *Psidium guajava* fruit in streptozotocin-induced diabetic rats. *Food and Chemical Toxicology*.

[9] Choi S., Hwang J., Park S. (2008). Fermented guava leaf extract inhibits LPS-induced COX-2 and iNOS expression in Mouse macrophage cells by inhibition of transcription factor NF-*κ*B. *Phytotherapy Research*.

[10] Wehbe H., Henson R., Meng F., Mize-Berge J., Patel T. (2006). Interleukin-6 contributes to growth in cholangiocarcinoma cells by aberrant promoter methylation and gene expression. *Cancer Research*.

[11] Kloek J. J., Marsman H. A., van Vliet A. K., Gouma D. J., van Gulik T. M. (2008). Biliary drainage attenuates postischemic reperfusion injury in the cholestatic rat liver. *Surgery*.

[12] Wuestefeld T., Klein C., Streetz K. L. (2005). Lack of gp130 expression results in more bacterial infection and higher mortality during chronic cholestasis in mice. *Hepatology*.

[13] El-Faramawy A. A. M., El-Shazly L. B. E., Abbass A. A., Ismail H. A. B. (2011). Serum IL-6 and IL-8 in infants with biliary atresia in comparison to intrahepatic cholestasis. *Tropical Gastroenterology*.

[14] DeMauro S. B., Kilpatrick L. E., Gerdes J. S., Abbasi S. (2012). Early inflammatory markers for prediction of cholestasis in very-low-birth-weight infants. *Neonatology*.

[15] Shi Y., Frost P., Hoang B., Benavides A., Gera J., Lichtenstein A. (2011). IL-6-induced enhancement of c-Myc translation in multiple myeloma cells: critical role of cytoplasmic localization of the RNA-binding protein hnRNP A1. *The Journal of Biological Chemistry*.

[16] Nabata T., Morimoto S., Koh E., Shiraishi T., Ogihara T. (1990). Interleukin-6 stimulates c-Myc expression and proliferation of cultured vascular smooth muscle cells. *Biochemistry International*.

[17] Bontempo P., Doto A., Miceli M. (2012). *Psidium guajava* L. anti-neoplastic effects: Induction of apoptosis and cell differentiation. *Cell Proliferation*.

[18] Georgiev P., Jochum W., Heinrich S. (2008). Characterization of time-related changes after experimental bile duct ligation. *British Journal of Surgery*.

[19] Yang H., Ramani K., Xia M. (2009). Dysregulation of glutathione synthesis during cholestasis in mice: molecular mechanisms and therapeutic implications. *Hepatology*.

[20] Quinn M., Ueno Y., Pae H. Y. (2012). Suppression of the HPA axis during extrahepatic biliary obstruction induces cholangiocyte proliferation in the rat. *American Journal of Physiology—Gastrointestinal and Liver Physiology*.

[21] Francis H., Glaser S., Ueno Y. (2004). CAMP stimulates the secretory and proliferative capacity of the rat intrahepatic biliary epithelium through changes in the PKA/Src/MEK/ERK1/2 pathway. *Journal of Hepatology*.

[22] Xia X., Francis H., Glaser S., Alpini G., LeSage G. (2006). Bile acid interactions with cholangiocyte. *World Journal of Gastroenterology*.

[23] Duncan J. S., Whittle M. C., Nakamura K. (2012). Dynamic reprogramming of the kinome in response to targeted MEK inhibition in triple-negative breast cancer. *Cell*.

[24] Kawata K., Kobayashi Y., Gershwin M. E., Bowlus C. L. (2012). The immunophysiology and apoptosis of biliary epithelial cells: primary biliary cirrhosis and primary sclerosing cholangitis. *Clinical Reviews in Allergy and Immunology*.

[25] Tischendorf J. J. W., Hecker H., Krüger M., Manns M. P., Meier P. N. (2007). Characterization, outcome, and prognosis in 273 patients with primary sclerosing cholangitis: a single center study. *American Journal of Gastroenterology*.

[26] Starzl T. E., Demetris A. J., van Thiel D. (1989). Liver transplantation (first of two parts). *The New England Journal of Medicine*.

[27] Hirschfield G. M., Heathcote E. J., Gershwin M. E. (2010). Pathogenesis of cholestatic liver disease and therapeutic approaches. *Gastroenterology*.

[28] Carey E. J., Lindor K. D. (2012). Current pharmacotherapy for cholestatic liver disease. *Expert Opinion on Pharmacotherapy*.

[29] Ko K. S., Peng J., Yang H. (2013). Animal models of cholangiocarcinoma. *Current Opinion in Gastroenterology*.

[30] Gong Y., Huang Z., Christensen E., Gluud C. (2007). Ursodeoxycholic acid for patients with primary biliary cirrhosis: an updated systematic review and meta-analysis of randomized clinical trials using Bayesian approach as sensitivity analyses. *American Journal of Gastroenterology*.

[31] Gutiérrez R. M. P., Mitchell S., Solis R. V. (2008). *Psidium guajava*: a review of its traditional uses, phytochemistry and pharmacology. *Journal of Ethnopharmacology*.

[32] Aronson D. C., de Haan J., James J. (1988). Quantitative aspects of the parenchyma-stroma relationship in experimentally induced cholestasis. *Liver*.

[33] Bauer M., Schuppan D. (2001). TGF*β*1 in liver fibrosis: time to change paradigms?. *FEBS Letters*.

[34] Cutroneo K. R. (2003). How is type I procollagen synthesis regulated at the gene level during tissue fibrosis. *Journal of Cellular Biochemistry*.

[35] Kwak H., Park M., Cho H. (2006). Transforming growth factor-*β*1 induces tissue inhibitor of metalloproteinase-1 expression via activation of extracellular signal-regulated kinase and Sp1 in human fibrosarcoma cells. *Molecular Cancer Research*.

[36] Copple B. L., Jaeschke H., Klaassen C. D. (2010). Oxidative stress and the pathogenesis of cholestasis. *Seminars in Liver Disease*.

[37] Yongvanit P., Pinlaor S., Bartsch H. (2012). Oxidative and nitrative DNA damage: key events in opisthorchiasis-induced carcinogenesis. *Parasitology International*.

[38] Lin C. Y., Yin M. C. (2012). Renal protective effects of extracts from guava fruit (*Psidium guajava* L.) in diabetic mice. *Plant Foods for Human Nutrition*.

[39] Janicke B., Hegardt C., Krogh M. (2011). The antiproliferative effect of dietary fiber phenolic compounds ferulic acid and p-coumaric acid on the cell cycle of Caco-2 cells. *Nutrition and Cancer*.

[40] Luo H., Daddysman M. K., Rankin G. O., Jiang B., Chen Y. C. (2010). Kaempferol enhances cisplatin's effect on ovarian cancer cells through promoting apoptosis caused by down regulation of cMyc. *Cancer Cell International*.

[41] Park S., Choi J. (2010). Inhibition of *β*-catenin/Tcf signaling by flavonoids. *Journal of Cellular Biochemistry*.

[42] Chen Z., Zeng H., Guo Y. (2010). MiRNA-145 inhibits non-small cell lung cancer cell proliferation by targeting c-Myc. *Journal of Experimental and Clinical Cancer Research*.

[43] van Breemen R. B., Pajkovic N. (2008). Multitargeted therapy of cancer by lycopene. *Cancer Letters*.

[44] Jordán M. J., Margaría C. A., Shaw P. E., Goodner K. L. (2003). Volatile components and aroma active compounds in aqueous essence and fresh pink guava fruit puree (*Psidium guajava* L.) by GC-MS and multidimensional GC/GC-O. *Journal of Agricultural and Food Chemistry*.

