# Supplementary figures and images for: Protective Effects of Guava Pulp on Cholestatic Liver Injury
Source: ISRN Hepatol. 2013 Nov 17;2013:601071. doi: 10.1155/2013/601071 (PMC4890915; doi:10.1155/2013/601071)

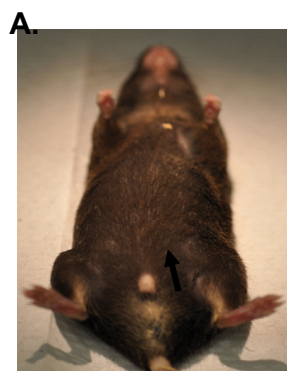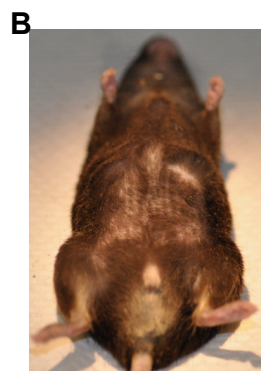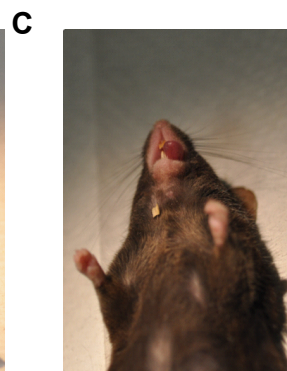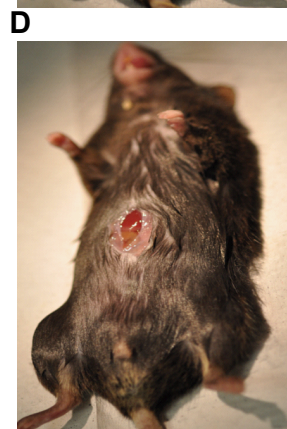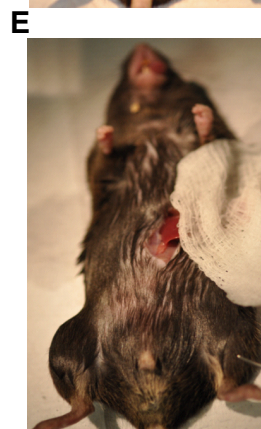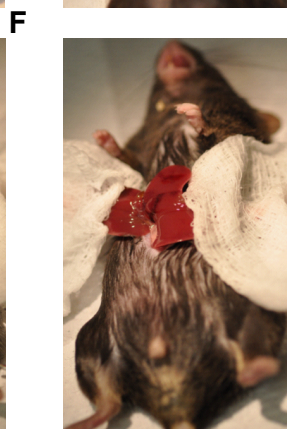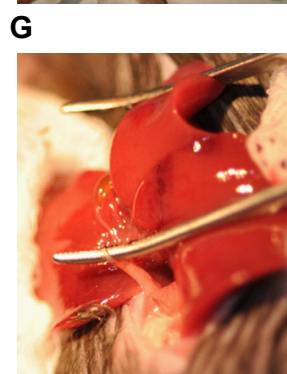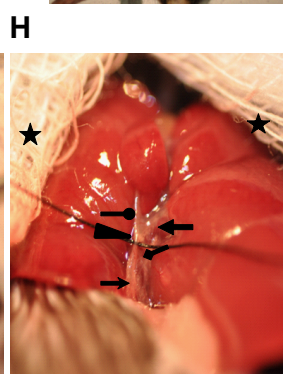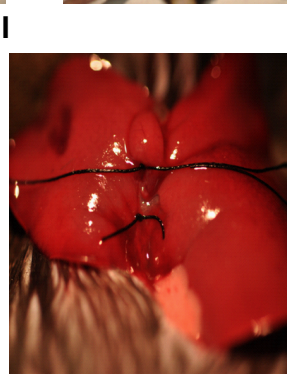

Supplement: Supplementary file 1 — Supplementary Figure: Procedure of LMBDL surgery. A) Ketamine (100 mg/kg/BW) with Xylazine (5 mg/kg/BW) was intraperitoneally injected into the lower right quadrant of the mouse. B) Hair was removed from the operative site. C) The mouth of the mouse was gently prying opened with forceps, and then the tongue was pulled out and held to the right side. D) The abdomen skin was cleaned by swabbing with 70% ethanol solution followed by povidone iodine solution to prevent bacterial infection during the LMBDL surgical procedure. Then, laparotomy was performed to expose the abdominal contents with a small pair of scissors. The incision about 3 cm long started at the midabdomen and ended at the xiphoid process. E) Two pieces of gauze were moistened (sterile 0.9% saline) and placed on the right side of the incision (left side of mouse). Three pieces of moistened cotton gauzes were used to lift the left lobe onto the skin of the left abdomen. F) The median lobe onto the skin of the right abdomen. G) The bile duct and associated structures were exposed. Upper margin of the right lobe was determined and the portal triad (bile duct, portal vein and hepatic artery) was revealed carefully. The key step was isolation of the hepatic bile duct between the mergence of the left and median lobe and the mergence of the right and caudate lobe. H,I) The isolated hepatic bile duct was ligated with a 6-0 silk suture. Since the fluxed passages of bile in median and left lobes (approximately 70% of the liver) were blocked, the ligated bile duct would soon be full of bile. After the isolated bile duct to the gall bladder was ligated, the gallbladder was removed to avoid cholecystitis. The peritoneum was closed with a 6-0 silk suture. [file 601071.f1.pdf]
